# Supramolecular Thixotropic Ionogel Electrolyte for Sodium Batteries

**DOI:** 10.3390/gels8030193

**Published:** 2022-03-20

**Authors:** Shipeng Chen, Li Feng, Xiaoji Wang, Yange Fan, Yubin Ke, Lin Hua, Zheng Li, Yimin Hou, Baoyu Xue

**Affiliations:** 1 Henan Institute of Chemistry, Henan Academy of Sciences, No. 56 Hongzhuan Road, Zhengzhou 450003, China; fblues@163.com (Y.F.); 18637162785@163.com (L.H.); lizheng0229@126.com (Z.L.); 2 Jiangsu Sunpower Co., Ltd., No 8 of Xingyuan Road, Huangqiao Industrical Park, Taixing 225400, China; fengli@jssanjie.com; 3 Guangdong-Hong Kong-Macao Joint Laboratory for Neutron Scattering Science and Technology, 1 Zhongziyuan Road, Dalang, Dongguan 523803, China; professorwxj@163.com (X.W.); keyb@ihep.ac.cn (Y.K.); 4 School of Chemical Engineering and Energy Technology, Dongguan University of Technology, Dongguan 523808, China

**Keywords:** low-molecular-weight gel, shear thinning, supramolecular interactions, self-assembly, thixotropic mechanism

## Abstract

Owing to the potential of sodium as an alternative to lithium as charge carrier, increasing attention has been focused on the development of high-performance electrolytes for Na batteries in recent years. In this regard, gel-type electrolytes, which combine the outstanding ionic conductivity of liquid electrolytes and the safety of solid electrolytes, demonstrate immense application prospects. However, most gel electrolytes not only need a number of specific techniques for molding, but also typically suffer from breakage, leading to a short service life and severe safety issues. In this study, a supramolecular thixotropic ionogel electrolyte is proposed to address these problems. This thixotropic electrolyte is formed by the supramolecular self-assembly of D-gluconic acetal-based gelator (B8) in an ionic liquid solution of a Na salt, which exhibits moldability, a high ionic conductivity, and a rapid self-healing property. The ionogel electrolyte is chemically stable to Na and exhibits a good Na^+^ transference number. In addition, the self-assembly mechanism of B8 and thixotropic mechanism of ionogel are investigated. The safe, low-cost and multifunctional ionogel electrolyte developed herein supports the development of future high-performance Na batteries.

## 1. Introduction

In the last few decades, lithium (Li)-ion batteries (LIBs) have been extensively explored due to their outstanding energy and power densities [1,2,3]. However, with the successful commercialization of LIBs, serious safety concerns about ignition or explosions are gradually emerging [4], and the global Li supply for LIBs is depleting [5,6,7,8,9,10]. Thus, alternatives to LIBs have become a hot topic [11]. Considering the high abundance, low costs, and relatively low atomic mass (Compared with Mg, Zn, etc.) of sodium, as well as its similar chemical properties to those of Li, sodium (Na)-ion batteries (NIBs) have recently become a research focus [12,13]. However, the performance of NIBs is still far inferior to LIBs, and it is imperative to further investigate electrode materials and electrolytes for NIBs [12,14, 15,16,17]. 

The type of electrolyte is key to achieving optimal NIBs, because an electrolyte acts as a medium for charge transfer in batteries, which determines the current density, time stability, and safety or reliability of batteries [18]. Typical organic solvents such as propylene carbonate and ethylene carbonate containing Na salts exhibit a high ionic conductivity, a wide electrochemical stability window, a relatively higher capacity, and a stable anode-passivation layer; however, their flammability and high vapor pressure lead to endless security problems [14,16,19]. In this regard, all-solid-state electrolytes have been considered as a promising alternative to address the safety issues because of nonflammability, high energy densities, and possible operation in a wide range of voltages and temperatures [20]; however, their intrinsic drawbacks, including low ionic conductivity, poor air compatibility, narrow electrochemical stability, and the grain-boundary resistance arising from the solid–solid interface, have not yet been resolved [16,18]. In recent years, considerable attention has been focused on the development of gel electrolytes, which exhibit good ionic conductivity due to the liquid-like ion-transport mechanism and good interfacial contact between electrodes and electrolytes due to mechanical softness and good wettability [18,21,22]. Moreover, gel electrolytes can effectively resist external mechanical stimulation and prevent electrolyte leakage [23,24,25]. Generally, gel electrolytes are more ideal electrolyte materials compared with their liquid and solid counterparts because of their safety, high ionic conductivity, and good electrochemical properties [26]. 

**Chart 1:**
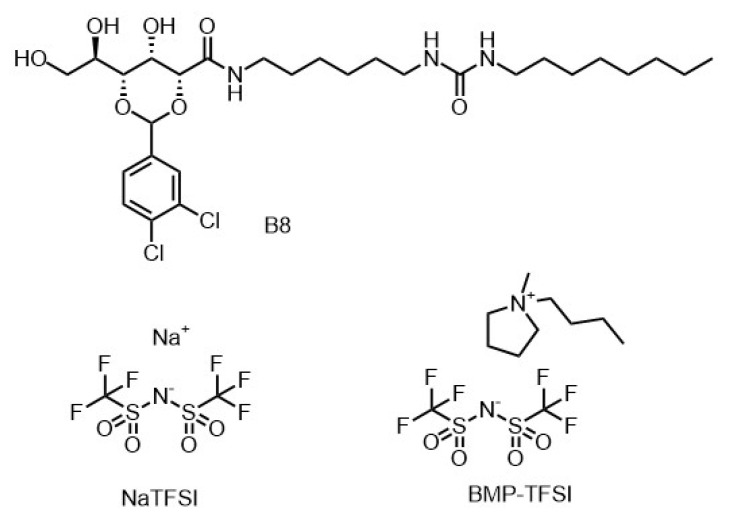
Chemical structures of B8, BMPTFSI, and NaTFSI.

In previous studies, we developed a series of D-gluconic acetal-based gelators that can harden a wide variety of ILs, the conductivity and rheological properties of the resultant ionogels were systematically investigated, and the application potential of several outstanding ionogels in flexible display, intelligent conductivity, and lubricants was explored [27,28]. Herein, a novel supramolecular thixotropic ionogel electrolyte for Na batteries is described. A room-temperature ionic liquid (IL), viz. butylmethylpyrrolidinium–bis-(trifluoromethylsulfonyl)imide (BMPTFSI, Chart 1), was selected as the electrolyte substrate because its cathodic stability limit (associated with the reduction of BMP^+^) is beyond the Na plating/stripping reaction, and TFSI^–^ can withstand a high potential (>5.0 V vs. Na) [29]; moreover, excellent properties of ILs such as non-flammability, a low vapor pressure, wide electrochemical stability, and high thermal stability guarantee the safety of electrolyte materials [30,31]. A D-gluconic acetal-based gelator (B8, Chart 1) was selected to gel the IL [27]. Fortunately, the resultant supramolecular ionogel electrolyte based on B8, BMPTFSI, and NaTFSI (Chart 1) exhibited unprecedented performance, including favorable ionic conductivity, good Na^+^ transference number (T_Na_^+^), high mechanical strength, chemical stability, thixotropy, and a rapid self-healing property, indicative of a novel intelligent gel electrolyte with application potential for sodium batteries. Thixotropy, as well as stimuli-responsive liquid-/solid-phase transitions, are significant for the molding of materials in several industrial processes, including paints, ceramic sols, and device assembly [32]. Moreover, the self-assembly and thixotropic mechanism of the supramolecular ionogel electrolyte were investigated. Addressing the shortage of lithium resources and bright prospect of NIBs, our studies on developing a multifunctional ionogel electrolyte provide some insights into the future development of high-performance supramolecular ionogel electrolytes for NIBs.

## 2. Results and Discussion

### 2.1. Preparation of the B8-BMPTFSI Ionogel and Insights into the Mechanism of Self-Assembly and Thixotropy

**Figure 1:**
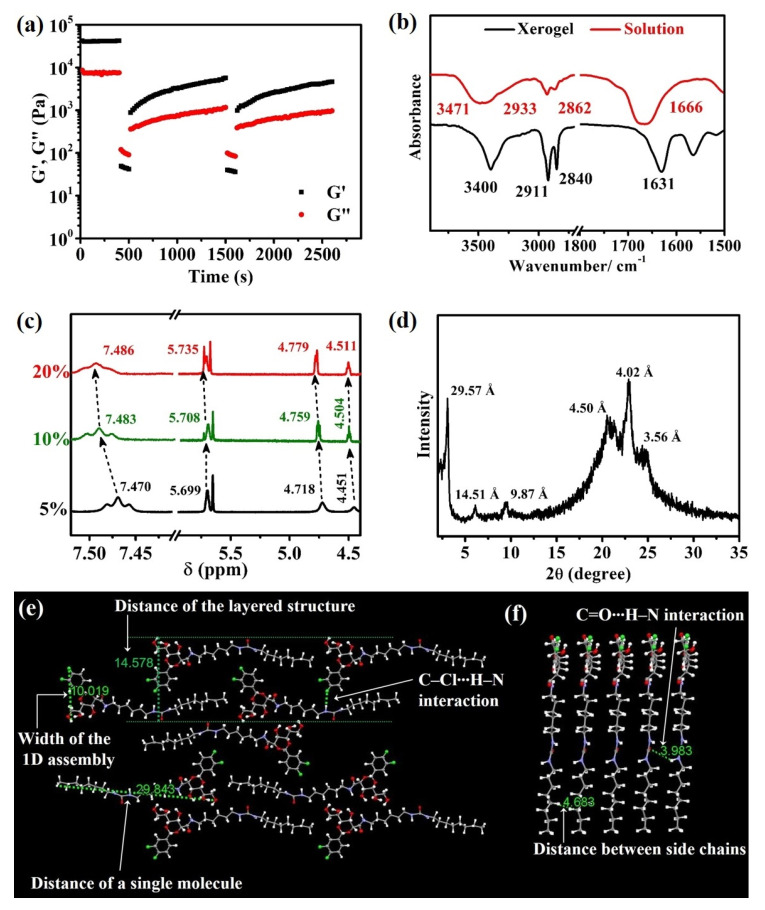
(**a**) Step-strain measurements of the B8-BMPTFSI ionogel (2%, w/v) over 2 cycles (with an alternating strain of 0.05 and 100% with 1 Hz at 25 °C); (**b**) FT-IR spectra of the B8 xerogel and DMF solution of B8; (**c**) changes in the ^1^H NMR shift of N-H and different O-H groups of B8 in BMPTFSI/DMSO-d_6_ at ratios (v/v) of 40:60 with the increase in the B8 concentration from 5% (w/v) to 20% (w/v); (**d**) XRD diffraction pattern of the B8-BMPTFSI ionogel (2%, w/v). The optimized self-assembly mode of B8 obtained by theoretical calculations (Gaussian 09 B3LYP method): (**e**) The length of the B8 molecule and the distance of a layered structure of B8, the width of the 1D assembly of B8, and the C-Cl‧‧‧H-N H-bonding interaction between B8 molecules; (**f**) the distance between the side chains of A8, the C=O…H-N interaction between A8 molecules. Color code: C = gray; H = white; O = red; N = blue; Cl = green.

To prepare a B8-BMPTFSI ionogel, an appropriate amount of B8 was added into a certain volume of BMPTFSI, which was stirred at 60 °C. After B8 was completely dissolved, the hot solution was cooled to 25 °C for 20 min, affording the B8-BMPTFSI ionogel. The critical gelation concentration (CGC) of the B8-BMPTFSI ionogel was 0.7% (w/v), and the ionogel was thermal-reversible, the sol–gel phase inversion temperature (Tg) of the 0.7% (w/v) B8-BMPTFSI ionogel was 76.4 °C. The B8-BMPTFSI ionogel exhibited hysteresis-free thixotropic behavior, and corresponding rheological data (Figure 1a) indicated that the gel can recover itself after damage by shear force, and that recovery can be repeated. 

The driving forces for the self-assembly of B8 were investigated (Figure 1b,c): The fourier transform infrared (FT-IR) spectrum of the B8 xerogel revealed that O-H and N-H stretching vibration bands overlap at 3400 cm^−1^, CH_2_ stretching vibration bands are observed at 2911 and 2840 cm^–1^, and the characteristic carbonyl stretching vibration band appeared at 1631 cm^−1^. Nevertheless, the corresponding stretching vibration bands of B8 in a DMF solution were blue-shifted, indicative of the participation of the O-H, N-H, and C=O groups in hydrogen bonding, as well as the presence of van der Waals (VDW) forces between the side chains of B8 [33]. ^1^H NMR spectral analysis of B8 at variable concentrations in DMSO-d_6_ was also conducted in the presence of BMPTFSI. At 5% (w/v) B8, the N-H signal of the amide group was observed at 7.470 ppm, N-H signals of the urea group were observed at 5.699 ppm, and different O-H signals were observed at 4.718 and 4.451 ppm. However, with the increase in the B8 concentration, all the above-mentioned signals shifted downfield, indicating that the protons of amide, urea, and hydroxyl groups are involved in hydrogen-bond formation [34].

X-ray diffraction (XRD) patterns of the B8-BMPTFSI ionogel (Figure 1d) were recorded to obtain information regarding the self-assembly of B8. Three clear reflection peaks corresponding to d-spacings of 29.57 Å, 14.51 Å, and 9.87 Å in a ratio of 1: 1/2: 1/3 were observed, respectively, suggesting the presence of a lamellar structure with a periodicity of 29.57 Å in the gel state [35]. The peak corresponding to a d-spacing of 3.56 Å is characteristic of the π-π stacking distance, indicative of the presence of π-π interactions between B8 molecules [36]. The peak with a d-spacing of 4.02 and 4.50 Å corresponded to the hydrogen-bonding distance of A8 molecules and the packing distance between the long alkyl chains of A8 molecules, respectively [37].

Attempts were made to prepare a B8 crystal to investigate the self-assembly mode of B8; however, all attempts were futile. Fortunately, however, the crystal of Z1, a precursor compound of G16 and A8, was obtained from an H_2_O/methanol (1:1) solution, and its single-crystal XRD data were collected (Figure S2, Table S1, and CIF S1): Assemblies of D-gluconic acetal fragments along the 1D direction were observed. Based on the single-crystal XRD data of Z1 and by theoretical calculation (Gaussian 09), the self-assembly mode of B8 was optimized and simulated (CIF S2). Figure 1e, f shows the calculated assembly mode of B8: The H-bonding interactions of polyhydroxy fragments and π···π interactions of chlorinated benzenes undoubtedly played a key role in the formation of the 1D assembly [27,37], and the two chlorine atoms on the benzene ring played a subtle role in supramolecular assemblies, which is well known to involve dispersive halogen interactions (halogen–arene and halogen–halogen interactions) [38,39]. In addition, H-bonding interactions of C=O···H-N between side chains were observed, which were also responsible for the 1D assembly of B8 (Figure 1f), the bond lengths of which were 3.983 Å, corresponding to a d-spacing of 4.02 Å in the XRD experiment (Figure 1d) [40]. C –Cl···H-N interactions (Figure 1e) [37,41] and VDW forces between side chains played a key role in the 3D assembly of B8. As shown in Figure 1e and f, the distance between side chains, the width of 1D assembly, the distance of the layered structure, and the distance of B8 molecule were 4.683 Å, 10.019 Å, 14.578 Å, and 29.843 Å, respectively, corresponding to d-spacings of 4.50 Å, 9.87 Å, 14.51 Å, and 29.57 Å in the XRD spectrum (Figure 1d). Although slight deviations were observed, it was sufficient to confirm the reliability of our calculation results.

In conclusion, the 1D self-assembly of B8 gelators depended on π-π stacking between benzene rings, hydrogen-bonding between polyol fragments, and C=O···H-N H-bonding interactions between side chains. The C-Cl···H-N interactions and VDW forces between side chains were responsible for the 3D self-assembly of B8 assemblies. In addition, the re-contact and re-assembly of the functional groups on the 1D assembly surface after mechanical damage is the essence of thixotropy [42,43].

### 2.2. Influence of Cations on the B8-BMPTFSI Ionogel

**Figure 2:**
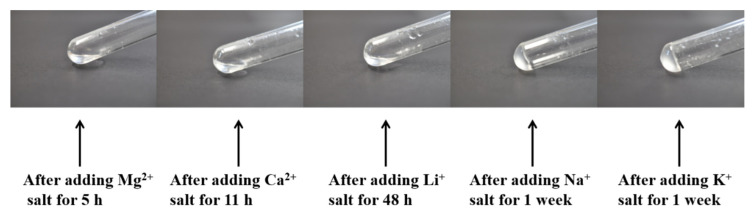
Influence of cations on the B8-BMPTFSI gel.

The internal network of supramolecular gels is based on physical interactions between molecules, which is sensitive to external stimuli, including charged ions. The influence of cations on the 2% (w/v) B8-BMPTFSI gel was tested by the addition of different salts with TFSI counter ions at a 1M concentration (Figure 2). The gel was completely destroyed after the addition of Mg^2+^ salt for 5 h, of Ca^2+^ salt for 11 h, and of Li^+^ salt for 48 h (mainly due to the destruction of intermolecular hydrogen bonds by cations) [44]. However, the gel maintained its original state for even 1 week after the addition of Na^+^ and K^+^. Obviously, the influence of cations on the B8-BMPTFSI gel decreased in the order of

K^+^ > Na^+^ > Li^+^ > Ca^2+^ > Mg^2+^.

The cation series above fitted perfectly into the Hofmeister series: The ions on the left tended to stabilize the native structure of a structural fragment and decrease its solubility, while those on the right tended to facilitate protein denaturation and increase solubility [45,46].

Fortunately, Na^+^ did not permit B8 to lose its ability to self-assemble in BMPTFSI, thereby laying the foundation for the preparation of the sodium-ion gel electrolyte of B8. Herein, NaTFSI was selected as a source of Na^+^ in the ionogel electrolyte, and CGCs of B8 required to gel the BMPTFSI solution at different NaTFSI concentrations were tested (Table 1). Clearly, with the increase in the sodium concentration of IL, the B8 concentration required to form gel also increased. However, with the increase in the NaTFSI and B8 concentrations, the conductivity of the resultant ionogel electrolyte decreased.

**Figure 3:**
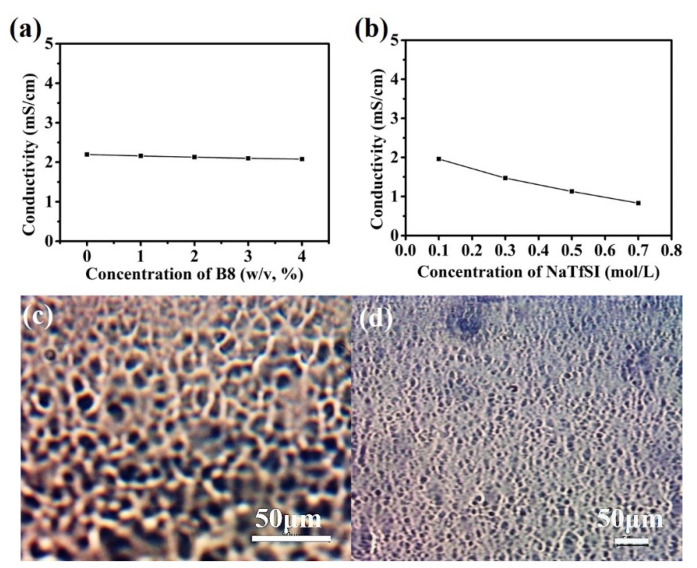
(**a**) Conductivities of the B8-BMPTFSI ionogel at different B8 concentrations; (**b**) Conductivities of the B8-NaTFSI-BMPTFSI ionogel (4% B8, w/v) at different NaTFSI concentrations, POM images of the (**c**) B8-BMPTFSI ionogel (4% B8, w/v) and (**d**) B8-BMPTFSI-NaTFSI ionogel (4% B8, w/v; 0.3 M NaTFSI).

To investigate the reason for the decrease in the conductivity of the gel electrolyte, a series of comparative tests were conducted. A marginal change in conductivities of B8-BMPTFSI ionogels at different B8 concentrations (from 0% to 4%, w/v) was observed (Figure 3a); however, with the increase in the NaTFSI concentration, the conductivity of the B8-BMPTFSI-NaTFSI ionogel decreased significantly (Figure 3b). Hence, the increase in the NaTFSI concentration directly leads to the decrease in electrolyte conductivity. As shown in Figure 3c,d, the morphologies of the B8-BMPTFSI gel (4% B8, w/v) and B8-BMPTFSI-NaTFSI gel (4% B8, w/v; 0.3 M NaTFSI) were visualized via polarizing optical microscopy (POM). Both of the abovementioned gels were characterized by a reticular network with several micropores, but the size of micropores of the B8-BMPTFSI-NaTFSI gel was far less than that of the B8-BMPTFSI gel. Moreover, according to the scanning electron microscope (SEM) images of xerogels (Figure S3), the abovementioned gels were also characterized by a reticular network, and the micropores of the xerogel of B8-BMPTFSI-NaTFSI gel were smaller than those of the B8-BMPTFSI gel. Smaller micropores were not favorable for ion movement, leading to the decrease in conductivity [47]. In conclusion, the addition of NaTFSI leads to a denser microstructure than the original gel [48], leading to the decreased conductivity of the gel electrolyte.

### 2.3. Practical Performance of the B8-BMPTFSI-NaTFSI Ionogel

**Figure 4:**
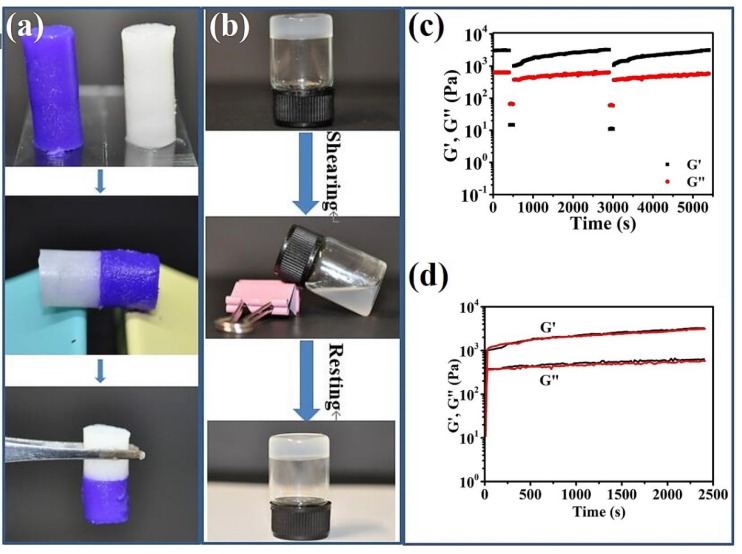
(**a**) Illustration of the self-healing property of the B8-BMPTFSI-NaTFSI gel (4% B8, w/v; 0.3 M NaTFSI). (**b**) Illustration of the thixotropic property of the B8-BMPTFSI-NaTFSI gel (3% B8, w/v; 0.1 M NaTFSI). (**c**) Step-strain measurements of the B8-BMPTFSI-NaTFSI gel (4% B8, w/v; 0.3 M NaTFSI) over 2 cycles (with an alternating strain of 0.05% and 100% with 1 Hz at 25 °C). (**d**) Overlaid zoom of the recovery of the B8-BMPTFSI-NaTFSI gel after each cycle. All colored gels are doped with crystal violet.

Outstanding mechanical characteristics and electrochemical performance are crucial factors for considering such a supramolecular ionogel for different applications. The B8-BMPTFSI-NaTFSI ionogel exhibited excellent self-healing and thixotropic properties. As shown in Figure 4a, the B8-BMPTFSI-NaTFSI ionogel (4% B8, w/v, 0.3 M NaTFSI) exhibited a self-standing property for possible molding into a column, and two pieces of the ionogel merged into one integrated block within minutes via their connection, illustrating the self-healing property sufficiently. In addition, the B8-BMPTFSI-NaTFSI ionogel (3% B8, w/v, 0.1 M NaTFSI) exhibited shear-thinning and recovered immediately after removing the shear force (Figure 4b). Step-strain measurement of oscillatory rheology for the B8-BMPTFSI-NaTFSI ionogel (4% B8, w/v; 0.3 M NaTFSI) was performed (Figure 4c): Complete recovery of the storage modulus was observed following strain-induced damage; moreover, the rate and extent of recovery were almost unchanged over two cycles of breaking and self-repairing (Figure 4d), indicative of the reversible and durable properties of the ionogel [49]. Further analysis of the rheological data showed a high G’/G” ratio (G’/G” = 5.36) and a high yield stress (14.4 Pa, as shown in dynamic stress sweep in Figure S4) for B8-BMPTFSI-NaTFSI ionogel (4% B8, w/v; 0.3M NaTFSI), indicative of a good mechanical stability of the ionogel [27,49,50].

**Figure 5:**
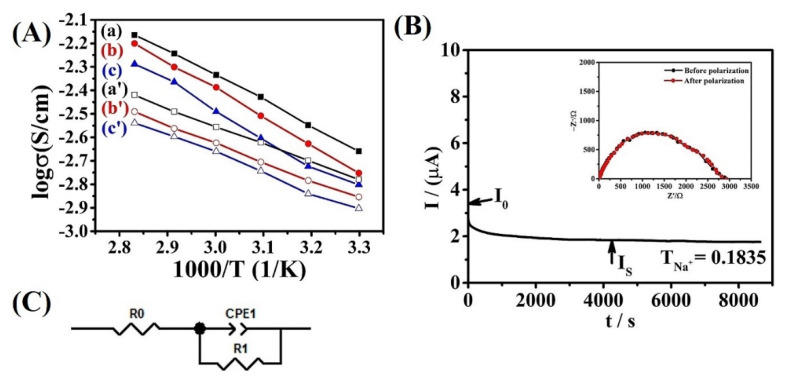
(**A**) Arrhenius plots for the conductivity of the B8-BMPTFSI gel (a) 3% B8, w/v, (b) 4% B8, w/v, and (c) 8% B8, w/v; B8-BMPTFSI-NaTFSI gel (a’) 3% B8 (w/v), 0.1 M NaTFSI, (b’) 4% B8 (w/v), 0.3 M NaTFSI, and (c’) 8% B8 (w/v), 0.5 M NaTFSI. (**B**) Chronoamperometry profiles of the symmetric Na/ B8-BMPTFSI-NaTFSI gel (4% B8, w/v, 0.3 M NaTFSI) /Na cell under a polarization voltage of 50 mV. Inset shows the corresponding electrochemical impedance spectroscopy (EIS) before and after polarization. (**C**) The model equivalent circuit derived from the Nyquist plots.

Figure 5A shows the temperature dependence of the conductivities of the B8-BMPTFSI and B8-BMPTFSI-NaTFSI ionogels. Although concentrations of B8 and NaTFSI were different, the relationship between the ion conductivity of all ionogels and 1000/T exhibited good linearity, indicative of a good fit into the classical Arrhenius equation [28]. In addition, the plots described in Figure 5A were fitted by the Vogel–Tammann–Fulcher (VTF) equation:

σ = σ_0_exp [ –B/ (T – T_0_)]

The result suggests that the migration of the ions in B8-BMPTFSI and B8-BMPTFSI-NaTFSI gels is similar to that of a viscous liquid [25]. The ionic conductivity of the B8-BMPTFSI-NaTFSI ionogel still achieved 10^−3^ S/m, albeit less than that of B8-BMPTFSI ionogel.

To evaluate the chemical stability of the B8-BMPTFSI-NaTFSI ionogel, aging experiments were performed on the ionogel; therefore, sodium metal was immersed into the ionogel and then placed in an inert environment at 50 °C for 30 days. After the B8-BMPTFSI-NaTFSI ionogel was in contact with the sodium metal at 50 / for 30 days, no clear color change was observed, and its state was not significantly different from that of the newly prepared ionogel (Figure S5a). In addition, ^1^H- NMR data indicated that the signals of the main functional groups of BMPTFSI and B8 of the aged ionogel do not change (Figure S5b–e), illustrating the excellent chemical stability of the B8-BMPTFSI-NaTFSI ionogel. Furthermore, electrochemical impedance spectroscopy (EIS) was employed to measure the T_Na_^+^ of the B8-BMPTFSI-NaTFSI gel (4% B8, w/v, 0.3 M NaTFSI) using a symmetrical Na/electrolyte/Na cell. Figure 5B represents the Nyquist plots for the ionogel electrolyte before and after polarization; a model equivalent circuit manifested from the Nyquist plots is shown as Figure 5C, in which R0 and R1 denotes the resistances to ionic and electronic transport, respectively [51]. The calculated T_Na_^+^ value of the ionogel was 0.1835, a satisfactory value [30].

Thus, a multifunctional ionogel electrolyte suitable for NIBs has been successfully developed. The performance parameters, including ionic conductivity, self-healing, and T_Na_^+^, of our ionogel electrolyte were compared with those of other ionogel electrolytes for NIBs (from 2010 to present, as shown in Table S2), the ionogel electrolyte of this work is the only self-healing ionogel electrolyte reported for Na batteries with high room temperature conductivity and good T_Na_^+^. However, a lot of work needs to be carried out before a high-performance sodium-ion battery can be successfully produced based on our ionogel, such as the compatibility study of the ionogel with different electrodes, the stability and efficiency study of the ionogel at different temperatures, etc.

## 3. Conclusions

In this study, a novel supramolecular ionogel electrolyte based on the D-gluconic acetal-based gelator with a favorable ionic conductivity, satisfactory Na^+^ transference number (T_Na_^+^), high mechanical strength, chemical stability, thixotropy, and rapid self-healing property was prepared. The introduction of NaTFSI induced a denser microstructure of the ionogel, leading to the decreased conductivity of the ionogel electrolyte; however, the resultant B8-BMPTFSI-NaTFSI ionogel still exhibited high conductivity. The B8-BMPTFSI-NaTFSI ionogel electrolyte exhibited excellent chemical stability to Na metal, which is a potentially safe electrolyte material for Na batteries. The thixotropic ionogel electrolyte effectively resisted external mechanical stimulation and prevented electrolyte leakage, thus prolonging the service life of the electrolyte material. In addition, the application of thixotropic nature to the electrolyte will realize its free molding in accordance to the shape of the conduction interface, which is difficult for several electrolytes. The self-assembly and thixotropic mechanisms of ionogel were also examined and described in detail. However, more work needs to be carried out to study the effect of ions on the self-assembly of organic low-molecular-weight gelators, so as to develop gelators with self-assembly ability in the presence of Li^+^ and Mg^2+^, and expand the application range of supramolecular ionogels in the field of metal-ion batteries. In total, the multifunctional thixotropic B8-BMPTFSI-NaTFSI ionogel developed exhibited immense potential for application in high-performance Na batteries, and our studies on self-assembly and thixotropic mechanism provided a direction for the future design of more functionalized supramolecular ionogel electrolytes.

## 4. Materials and Methods

### 4.1. Materials 

D-Gluconic acid, 3, 4-dichlorobenzaldehyde, 1, 6-hexanediamine, and 4-dimethylaminopyridine (DMAP) were purchased from Aladdin Bio-Chem Technology Co., Ltd. (Shanghai, China). Octyl isocyanate was purchased from Shanghai Macklin Biochemical Co., Ltd. BMPTFSI was purchased from Lanzhou Greenchem ILS, LIPC, and CAS (Lanzhou, China). The chemical reagents were commercially available and directly utilized without further purification.

### 4.2. Synthesis

A8: Synthetic route of B8 is shown in Figure S1. Precursor B6 was synthesized according to the method reported previously [52]. First, 3.9 g (8.72 mmol) of B6 was dissolved in 40 mL of anhydrous DMSO at room temperature and then cooled to 0 °C followed by the dropwise addition of 2 mL (11.34 mmol) of octyl isocyanate. Then, the reaction mixture was warmed to room temperature and stirred vigorously for 2 h, followed by pouring it into 200 mL of distilled water under vigorous stirring. A large amount of a white solid precipitated, which was collected by filtration. The filter cake was three times washed with distilled water. After drying, B8 was obtained as a white powder. Yield: 4.24 g (6.98 mmol, 80%).

### 4.3. Chemical Characterization

2, 4-(3, 4-dichloro) Benzylidene-N-(6- (3-octylureido) hexyl)-D-gluconamide (B8) 

^1^H NMR (400 MHz, DMSO-d6, 25 °C): δ7.89 (d, 1H), 7.67 (d, 1H), 7.55 (dd, J = 8.9, 1.4Hz, 1H), 7.47 (t, J = 6.1Hz, 1H), 5.68−5.74 (m, 2H), 5.67 (s, 1H), 4.73 (t, J = 7.4Hz, 2H), 4.46 (t, J = 5.8Hz, 1H), 4.35 (d, 1H), 4.00 (d, 1H), 3.76 (dd, J = 8.9, 1.3Hz, 1H), 3.60−3.67 (m, 1H), 3.52−3.58 (m, 1H), 3.38−3.45 (m, 1H), 3.06−3.15 (m, 2H), 2.89−2.97 (m, 4H), 1.28−1.46 (m, 4H), 1.23 (s, 16H), 0.85 (t, J = 6.8Hz, 3H). ^13^C NMR (100 MHz, C_2_D_6_O, 25 °C): δ169.2, 159.6, 138.2, 132.6, 131.8, 129.9, 128.8, 126.4, 99.1, 80.6, 79.3, 69.2, 62.8, 62.4, 39.7, 39.4, 38.6, 31.7, 30.2, 29.9, 29.2, 29.1, 26.7, 26.1, 22.4, 13.3. HRMS (ESI): m/z calcd. for C_28_H_45_Cl_2_N_3_O_7_Na^+^ [M + Na] ^+^ 628.2527, found 628.2526. Mp: 179−180 °C. All the spectra of B8 are shown in Figure S6–S8.

### 4.4. Instrumentation

Gelation tests were investigated by the typical tube-inversion method. The pre-weighed gelator in a certain solvent in a sealed test tube (inner diameter: 13 mm) was heated until its dissolution, then the resultant solution was cooled to room temperature. The test tube was inverted, and gelation was confirmed when a homogeneous substance was formed with no gravitational flow. The heating temperature selected to dissolve a certain gelator must be less than its melting point to prevent compound deterioration. The CGC is defined as the minimum amount of a gelator required to immobilize 1 mL of solvent. The Tg was determined by the “falling ball method” described previously [27]. The xerogel was prepared by freeze-drying following solvent exchange [28]; the ionogel was immersed into water for 5 days, and the water was refreshed every 5 h; solvent exchange was conducted at room temperature without stirring and ultrasonication, etc.; and the xerogel was obtained by subsequent freeze-drying step. 

The following experiments and tests were conducted at room temperature and a humidity of 50% unless specified otherwise. Rheological studies of ionogels were conducted using a rheometer (Anton Paar Physica MCR 301) equipped with a plate (15 mm diameter). The gels were equilibrated at 25 °C between the plates that were adjusted to a gap of 0.5 mm. The frequency sweep at a constant strain of 0.05% was obtained from 0.1 to 100 rad/s. Strain sweep was performed in the 0.01−150% range at a constant frequency (1 Hz). Time sweep was conducted to observe the recovery property of the gel. First, a constant strain of 0.05% was applied on the sample. Then, a constant strain that was sufficient to break the gel was applied, followed by the application of a constant strain (0.05%) again. The storage modulus G’ and the loss modulus G” of the sample were recorded as functions of time in this experiment.

All NMR spectra were recorded on a Bruker DPX 400 MHz spectrometer. Mass spectra were recorded on a TOF-QII high-resolution mass spectrometer. Morphologies of the thin gel samples on a glass plate were investigated by polarizing optical microscopy (POM, Nikon Eclipse 50i POL). The morphologies of the xerogels were obtained by a Hitachi S-4800 SEM instrument operating at 3–5 kV; the xerogels were coated with a thin layer of Au before the experiment. FT-IR spectra were recorded on an FTS 3000 spectrometer with KBr pellets. Powder X-ray diffraction (XRD) patterns were recorded on a Bruker D8-S4 instrument (Cu Kα radiation, λ = 1.546 Å). The d spacing values were calculated by Bragg’s law (nλ = 2d sin θ). 

Theoretical calculation was performed by Gaussian 09, and all of the reported structures were optimized by the density functional theory (DFT) using the B3LYP functional with the 6-31 G (d, p) basis set. Based on the (B3LYP/ 6-31 G (d, p)) optimized geometries, the energy results were further refined by single-point energy calculation at the B3LYP*/ 6-31 G (d, p) level of theory.

Conductivities of the ionogels were measured using a conductivity meter (DDS-307A, Shanghai INESA Scientific Instrument Co., Ltd., China). The electrode was immersed into the ionogel at a temperature above the Tg, followed by cooling at 25 °C for gelation. Conductivities at different temperatures were determined, and the temperature was controlled using a thermostatic heater. The Na^+^ transference number (T_Na_^+^) of the B8-BMPTFSI-NaTFSI gel was determined by a DC polarization method combined with impedance spectroscopy [30]. A small polarization (ΔV, 50 mV) was applied to a symmetrical Na/electrolyte/Na cell, and then the initial current (I_0_) and steady-state current (I_S_) were measured. The initial (R_0_) and final (R_S_) resistances of the passivation layers on the Na electrodes were also examined. T_Na_^+^ can be calculated according to

T_Na_^+^ = I_S_ (ΔV – I_0_R_0_)/I_0_ (ΔV – I_S_R_S_).

## Figures and Tables

**Table 1:** CGCs of B8 required to gel the BMPTFSI solution of different NaTFSI concentrations, and the corresponding conductivity of gel (σ).

NaTFSI Concentration	B8	
	CGC (w/v, %)	σ (mS/cm)
0.1M	3	1.90
0.3M	4	1.43
0.5M	8	0.99

## Data Availability

The data presented in this study are available on request from the corresponding author.

## Supplementary Materials

The following supporting information can be downloaded at: https://www.mdpi.com/article/10.3390/gels8030193/s1, Figure S1: Synthetic route of B8; Figure S2: Unit cell of the co-crystal of Z1 and H_2_O along the (a) a-axis, (b) b-axis, (c) c-axis. Color code: C = gray; H = white; O = red. (d) The chemical formula of Z1; Table S1: Crystallographic details of the co-crystal of Z1 and H_2_O; Table S1: Crystallographic details of the co-crystal of Z1 and H2O; Table S2: Performance parameters of ionogels for Na batteries; Figure S3: SEM images of the xerogels of (a) B8-BMPTFSI and (b) B8-BMPTFSI-NaTFSI ionogels; Figure S4: (a) Dynamic frequency sweep measurements and (b) Dynamic stress sweep measure-ments for B8-BMPTFSI-NaTFSI gel (4% B8, w/v; 0.3M NaTFSI); Figure S5: (a) B8-BMPTFSI-NaTFSI ionogel (4% B8, w/v, 0.3 M NaTFSI) (left), B8-BMPTFSI-NaTFSI ionogel (4% B8, w/v, 0.3 M NaTFSI) kept in inert environment at 50 °C for 30 days with Na (right); (b) ^1^H-NMR data of BMPTFSI of newly prepared ionogel; (c) ^1^H-NMR data of BMPTFSI of ionogel kept in inert environment at 50 °C for 30 days with Na; (d) ^1^H-NMR data of active groups of B8 in newly prepared ionogel; (e) ^1^H-NMR data of active groups of B8 in ionogel kept in inert environment at 50 °C for 30 days with Na; Figure S6–S8: ^1^H-NMR, ^13^C-NMR, and HRMS spectra of B8; CIF S1: Single-crystal XRD data of Z1; CIF S2: The self-assembly mode of B8.
