# Supplementary material for: Supramolecular Thixotropic Ionogel Electrolyte for Sodium Batteries
Source: Gels. 2022 Mar 20;8(3):193. doi: 10.3390/gels8030193 (PMC8953603; doi:10.3390/gels8030193)
Supplement: Supplementary file 1 [file gels-08-00193-s001.zip › gels-1633103-supplementary.pdf]

gels

MDPI

Supplementary Materials

Supramolecular Thixotropic Ionogel Electrolyte for Sodium Batteries

Shipeng Chen [1,*], Li Feng [2], Xiaoji Wang [3,4], Yange Fan [1], Yubin Ke [3], Lin Hua [1], Zheng Li [1], Yimin Hou [1,*] and Baoyu Xue [1,*]

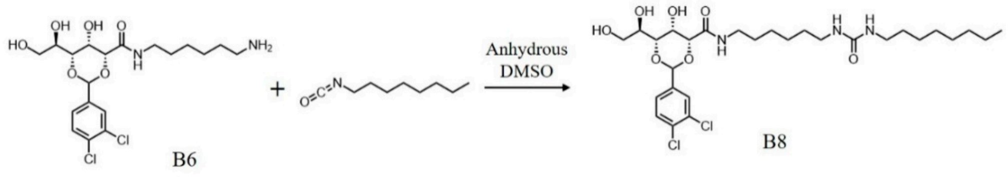

Figure S1. Synthetic route of B8.

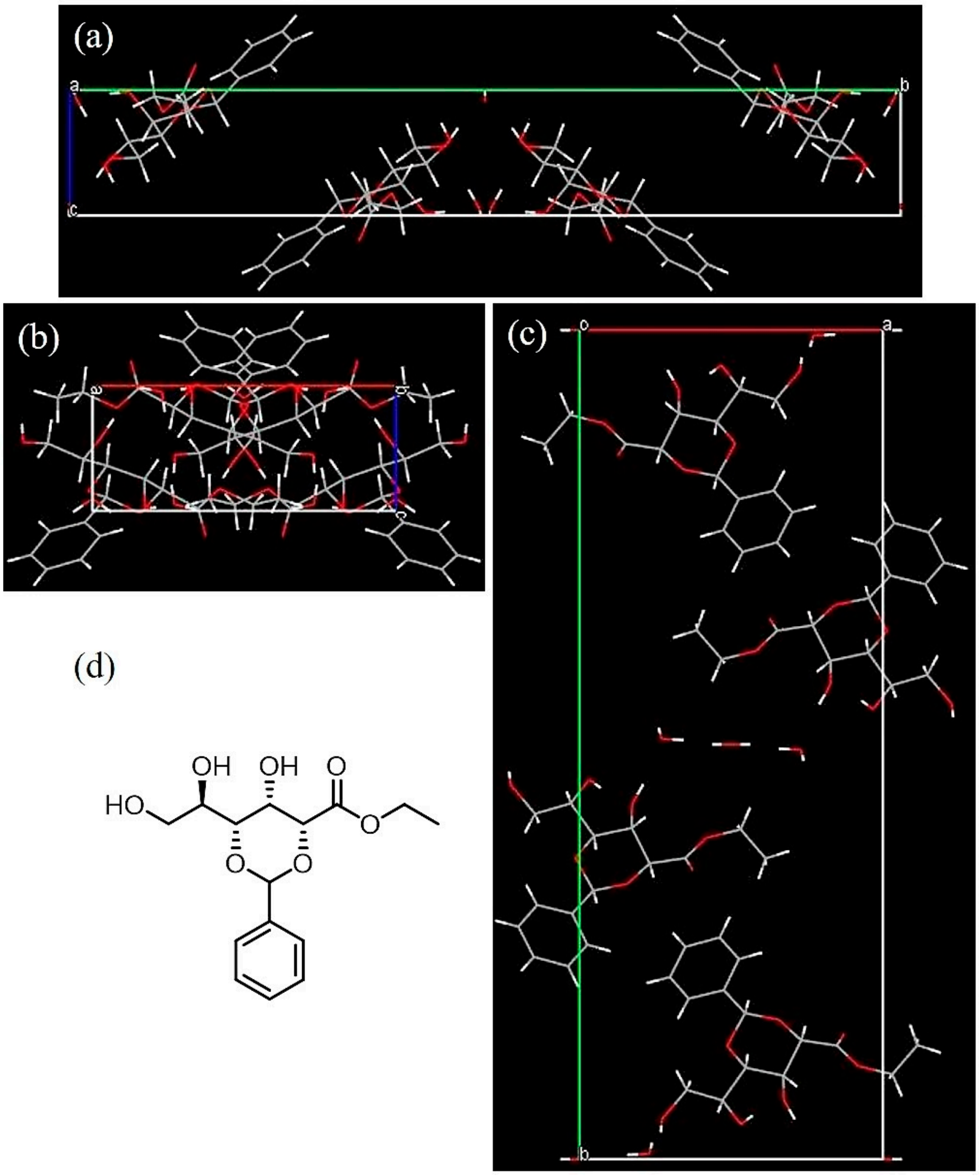

Figure S2. Unit cell of the co-crystal of Z1 and H_2O along the (**a**) a-axis, (**b**) b-axis, (**c**) c-axis. Color code: C = gray; H = white; O = red. (**d**) The chemical formula of Z1.

Table S1. Crystallographic details of the co-crystal of Z1 and H_2O.

**CCDC No.**	1960998
**Formula**	$C_{15}H_{20}O_7$
**Formula weight**	692.69
**Crystal system**	Orthorhombic
**Space group**	P 21 21 2
**T, K**	293
**λ (Cu Kα), Å**	1.54184
**a, Å**	11.3217(3)
**b, Å**	30.9860(7)
**c, Å**	4.6792(1)
**α, °**	90
**β, °**	90
**λ, °**	90
**V, Å^3**	1641.53(7)
**Z**	2
**Dx, g cm^{-3}**	1.401
**μ, mm^{-1}**	0.973
**F (000)**	740.0
**GOF**	1.068
**R1**	0.0522
**wR2**	0.1484

Table S2. Performance parameters of ionogels for Na batteries.

Year	Electrolyte ingredients	Ionic conductivity	Self-healing	T_{Na^+}	Ref.
2010	[P(VDF-HFP) + 0.5 M EMITf/NaCF3SO3]	5.74 × 10^{-3} S/cm at 27 °C	—	0.23	1
2015	[Silica fumed powder + 0.3 M $NaNTf_2/C_4mpyrNTf_2$]<br>[PMMA + 0.3 M $NaNTf_2/C_4mpyrNTf_2$]	1.1 × 10^{-3} S/cm at rt<br>7 × 10^{-4} S/cm at rt	—<br>—	—<br>—	2
2016	[(PEO + 10 wt.% NaMS) + 60 wt.% BMIM-MS]	1.05 × 10^{-4} S/cm at 30 °C	—	0.46	3
2016	[NaTFSI$(PEO)_9$ + 20 wt.% Pyr_{13}TFSI]	10^{-4} S/cm at 20 °C	—		4
2016	[(P(VDF-HFP) + 0.5 M NaTf/EMITf) + 5 wt.%Al_2O_3]<br>[(P(VDF-HFP) + 0.5 M NaTf/EMITf) + 5 wt.% $NaAlO_2$]	6.3 – 6.8 × 10^{-3} S/cm at rt<br>5.5 – 6.5 × 10^{-3} S/cmat rt	—<br>—	0.27<br>0.42	5
2017	[$(PEO_{20}/NaClO_4)$ + 5 wt.% SiO_2 +70 wt.% Emim FSI]	1.3 × 10^{-3} S/cm at rt	—	0.61	6
2017	[PEO/$NaClO_4$ + 30 wt.% 1-butyl-3-methylimidazolium thiocyanate]	5 × 10^{-4} S/cm at rt	—	—	7
2017	[P(VDF-HFP)/$NaN(CF_3SO_2)_2$]/Mono cationic ionic liquids(70:30, w/w)	2.2 × 10^{-4} S/cm at rt	—	0.1 – 0.5	8
2017	[PVC + Na[FSA]/[C_2C_1im][FSA]]	5.6 × 10^{-3} S/cm at 45 °C	—	—	9
2020	[SBVI/MPC/TFEMA + 0.5M NaTFSI/BMP TFSI]	1.6 × 10^{-3} S/cm at rt	—	0.19	10
2020	[SBA-15/ P(VDF-HFP) + NaTFSI/ PY_{13}FSI]	2.48 × 10^{-3} S/cm at 30 °C	—	0.37	11

2021	[m-PDMS/MTMS/HCOOH + 0.5M NaFSI/ C_4mpyrTFSI]	1 × 10^{-3} S/cm at rt	—	—	12
2021	[P(VDF-HFP)/TiO_2 + 0.5 M $NaCF_3SO_3$/BMImCF_3SO_3]	4 × 10^{-4} S/cm at rt	—	0.27	13
2022	[D-gluconic acetal-based gelator + 0.3 M NaTFSI /BMPTFSI]	1.43 × 10^{-3} S/cm at rt	Yes	0.1835	This work

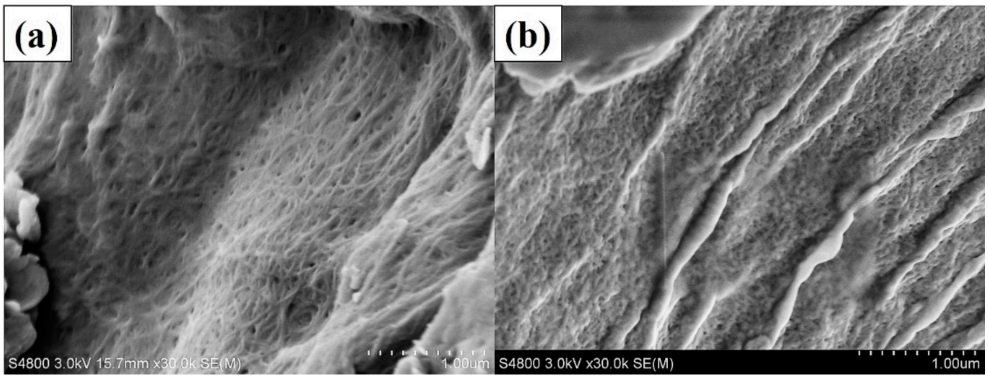

Figure S3. SEM images of the xerogels of (**a**) B8-BMPTFSI and (**b**) B8-BMPTFSI-NaTFSI ionogels.

The difference in morphology between SEM and POM images may be related to the preparation of xerogel; during solvent exchange and freeze-drying, the microscopic morphology of the gel is easily damaged, and the gelator originally dispersed in the solvent will also crystallize, resulting in the change of the original morphology.

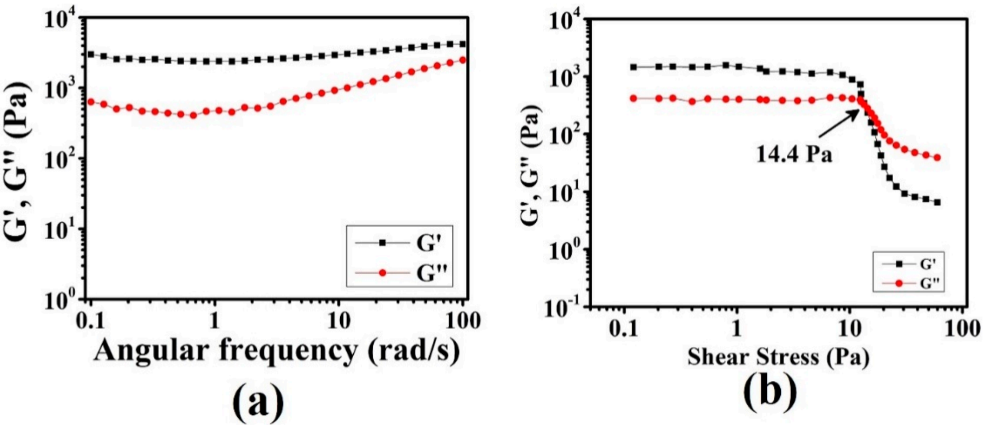

Figure S4. (**a**) Dynamic frequency sweep measurements and (**b**) Dynamic stress sweep measurements for B8-BMPTFSI-NaTFSI gel (4% B8, w/v; 0.3M NaTFSI).

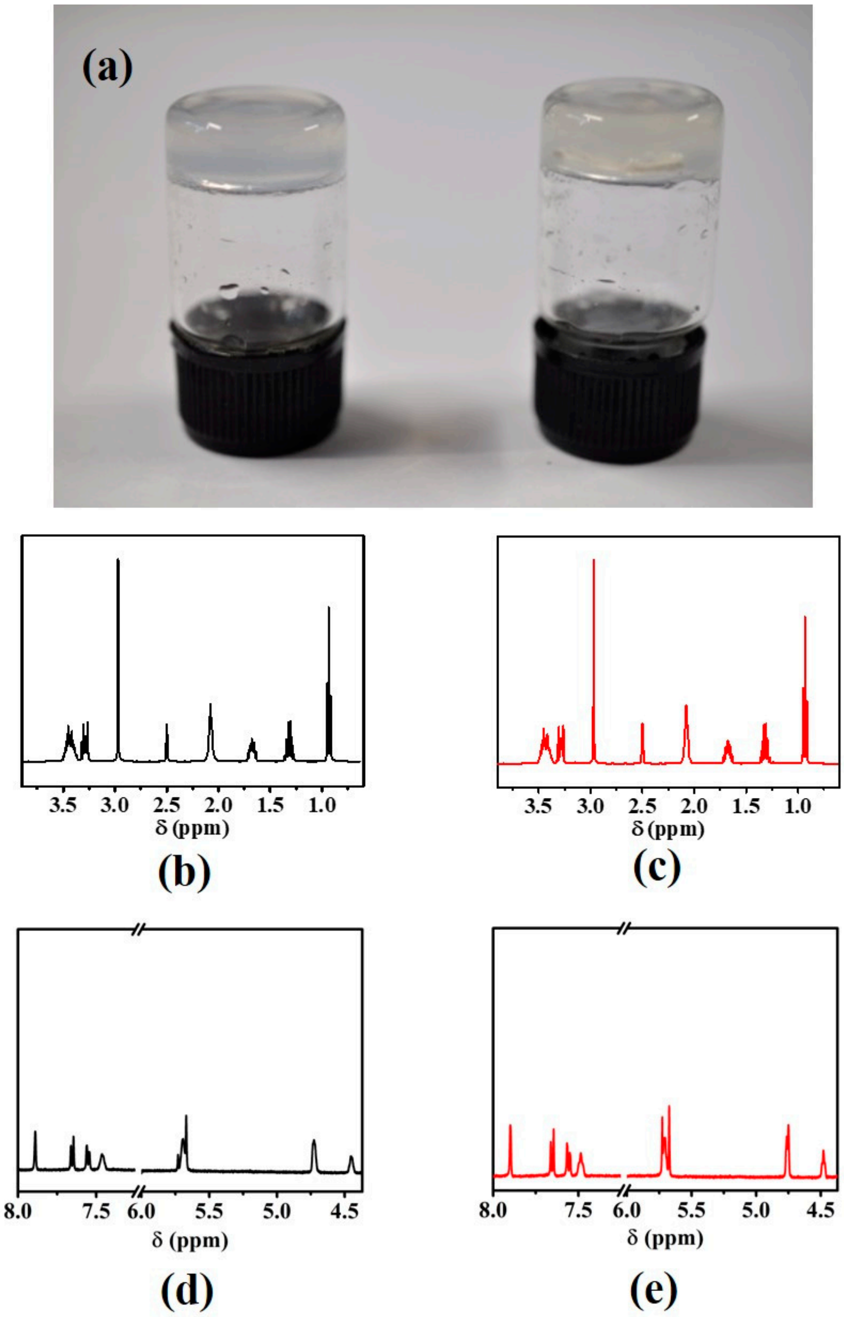

Figure S5. (**a**) B8-BMPTFSI-NaTFSI ionogel (4% B8, w/v, 0.3 M NaTFSI) (left), B8-BMPTFSI-NaTFSI ionogel (4% B8, w/v, 0.3 M NaTFSI) kept in inert environment at 50 °C for 30 days with Na (right); (**b**) ^{1}H-NMR data of BMPTFSI of newly prepared ionogel; (**c**) ^{1}H-NMR data of BMPTFSI of ionogel kept in inert environment at 50 °C for 30 days with Na; (**d**) ^{1}H-NMR data of active groups of B8 in newly prepared ionogel; (**e**) ^{1}H-NMR data of active groups of B8 in ionogel kept in inert environment at 50 °C for 30 days with Na.

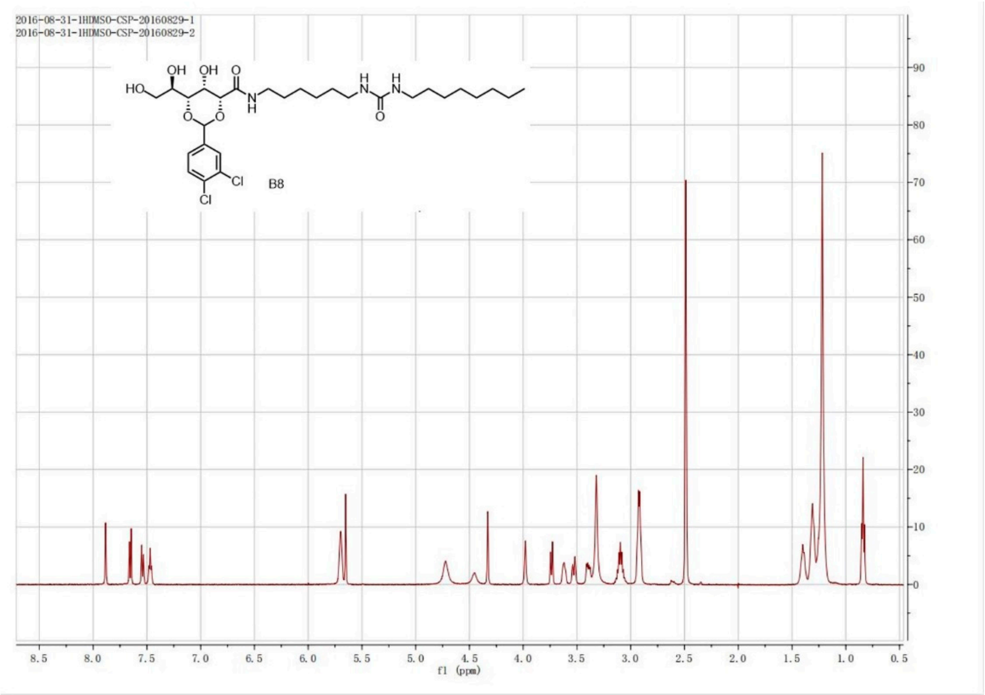

Figure S6. ^{1}H NMR (400MHz) Spectra of B8 in DMSO-d_6.

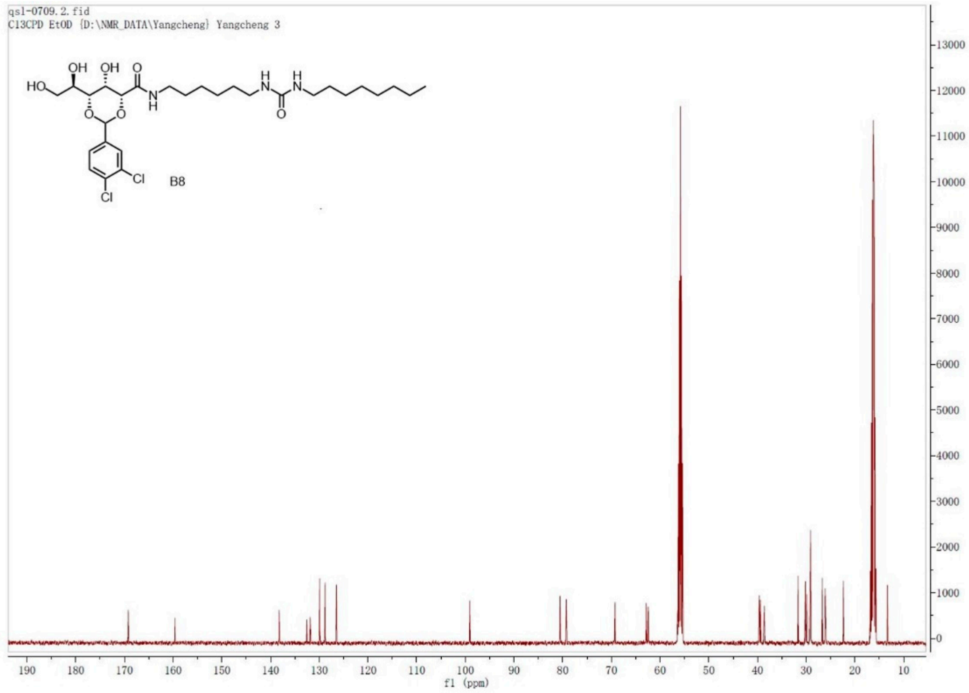

Figure S7. ^{13}C NMR (100MHz) Spectra of B8 in C_2D_6O.

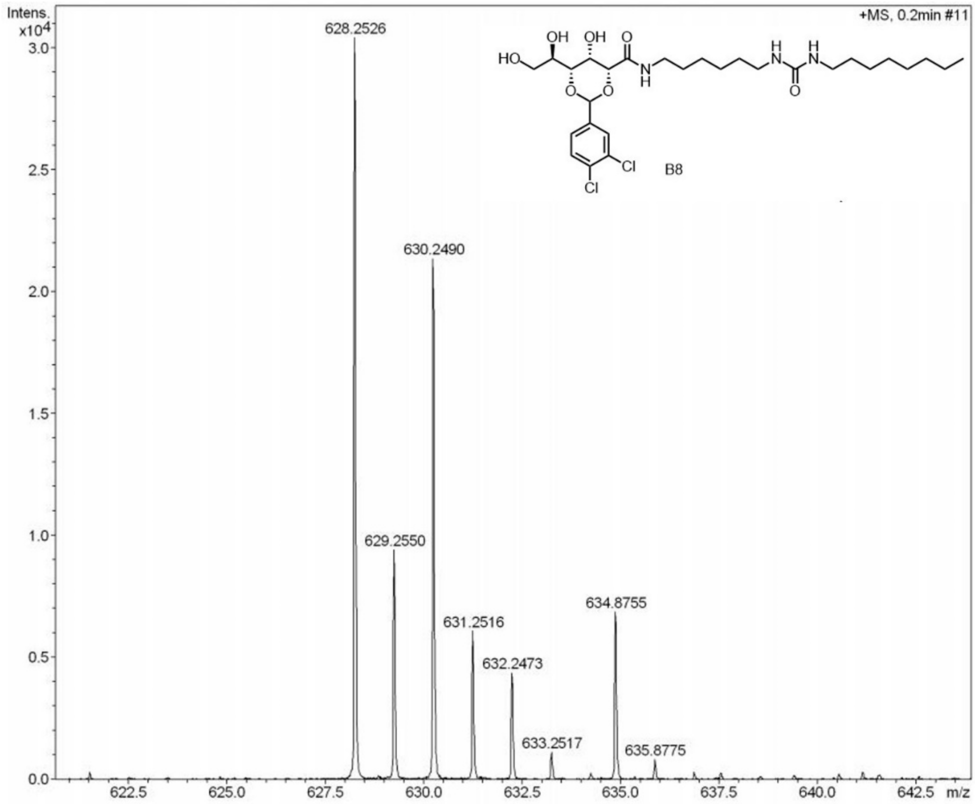

Figure S8. HRMS Spectra of B8 in DMF.

Supporting CIF S1, 2: Single-crystal XRD data of Z1 and the self-assembly mode of B8.
